# Hepatitis B Vaccination Rate in Patients with Diabetes: Assessment of Racial and Socioeconomic Disparity

**Published:** 2016-12-30

**Authors:** Ayse Aytaman, Nwakile Ojike, Samantha Zizi, SR Pandi-Perumal, Ismet Lukolic, Amit Bhanvadia, Felix Nwamaghinna, Haroon Kamran, Alla Akivis, Olusegun Bankole, Moro O Salifu, Samy I McFarlane

**Affiliations:** 1 VA NY Harbor Healthcare System, Brooklyn, NY, USA; 2 Department of Population Health, NYU School of Medicine, NYU Langone Medical Center, New York, USA; 3 Somnogen Canada Inc, Toronto, Ontario, CANADA; 4 Department of Medicine, State University of New York-Downstate Medical Center, Kings County Hospital Center, Brooklyn, NY 11203, USA; 5 Department of Family Medicine, State University of New York-Downstate Medical Center, Kings County Hospital Center, Brooklyn, NY 11203, USA

## Abstract

**Introduction:**

Less hygienic use of blood glucose monitoring equipment such as blood glucose meters, lancets, finger stick devices or other diabetes-care equipment such as syringes or insulin pens by self-administration often exposes the diabetic patient to Hepatitis B infection. This study evaluates hepatitis B vaccination among individuals with diabetes.

**Methods:**

The study used data from the 2000–2013 National Health Interview Survey (NHIS). Vaccination rates among adult individuals with diabetes of various ethnic backgrounds was accessed and compared using chis-square tests. Multivariable logistic regression model was used to compare factors affecting hepatitis B vaccination among individuals with diabetes.

**Results:**

The crude rate of diabetes in this population was 5.4%. The rate of vaccination among individuals with diabetes differed across racial groups (Asians 31.8% vs. blacks 30.7%; and whites 26.5%; p<0.01). After multivariate regression, the leading factors affecting hepatitis B vaccination included Age (40–60 years) (OR=0.51, 95% CI=0.47–0.57, p<0.01), lack of college education (OR=0.71,95% CI=0.64–0.79, p<0.01), foreign birth (OR=0.83, 95% CI=0.72–0.95, p<0.01), and Hispanic ethnicity (OR=0.88, 95% CI=0.78–1.00, P<0.05).

**Conclusion:**

Social and economic factors-education, insurance status, age, poverty level, and place of birth affect rates of vaccination among individuals with diabetes.

## Introduction

Recent evidence show that hepatitis B infection occurs more frequently among individuals with type 2 diabetes than the general population [1–3]. The odds for developing acute hepatitis B for individuals with diabetes is said to be as much as twice that for non-diabetics. The higher rates may not only be related to traditional sources of exposure such as blood transfusion, unsafe sex, unsterile surgery, intravenous drug abuse or other conditions involving improper handling of body fluids. From several outbreaks, hepatitis B infection among diabetics has also shown to be very often associated with poor hygiene standards surrounding the use of blood glucose monitoring equipment such as glucometers, lancets, or other diabetes-care equipment such as syringes and insulin pens either by patient self-administration or by caregiver administration at home, residential facilities, or hospitals [1,4].

The annual costs of managing type-2 diabetes and diabetes related illnesses is burdensome [5]. Superimposed hepatitis infection would substantially increase the burden on healthcare resources. The vaccination of all adults with diabetes aged 19–60 years is said to be moderately cost-effective, and forms the basis of CDC recommendations for this cohort [6–8]. However, it is unclear at present time of what the value of hepatitis B vaccination for diabetic individuals sixty years or older.

Some epidemiological studies suggest social factors including racial, knowledge, and geographic disparities; poverty, and inadequate access to health care have helped sustain the morbidity surrounding chronic hepatitis B infections [9–11]. The prevalence of hepatitis B infection is disproportionately high among Asian and African American populations [12,13].

The aim of the study is to compare the rates of hepatitis B vaccination among racial, demographic categories. A secondary objective was to evaluate the hepatitis B vaccination rates for various co-morbid conditions.

## Methods

Data Sources: Data for the study was taken from the 2000–2013 National Health Interview Survey (NHIS). The NHIS is a multipurpose national health survey conducted by the National Center for Health Statistics (NCHS) at the Centers for Disease Control and Prevention (CDC) and is designed to provide information about a wide range of health topics for the non-institutionalized US household population. Data was collected by trained interviewers with the U.S. Census Bureau who administer the survey during visits to selected households and by phone interview to administer the survey. The survey uses multistage, cluster sampling, with weights that are representative of the US adult population. All analyses performed in this study utilized weighted statistics based on the final weights provided with the NHIS data sets. These weights represent a product of weights for corresponding units computed in each of the sampling stages. Details on sample design can be found in Design and Estimation for the National Health Interview Survey 1995–2005, 2006–2013 [14].

The main factor evaluated in the study was hepatitis vaccination. Survey participants (SPs) gave answers to the survey question “Have you EVER received the hepatitis B vaccine?” Answers were recorded in a binary form as yes or no. Diabetes was categorized as yes or no based on self-reports. Age was recorded in whole numbers from 0–120 years as of their last birthday. The study focused on adults of 20–59 years. Age was further categorized into 20–39 years, and 40–59 years.

Differences in composition and distribution of categorical and continuous were assessed. χ2 test was employed to assess differences in categorical variables. A logistic regression model was used to determine associations between hepatitis B vaccine use and racial groups, demographic characteristics and co-morbid medical conditions among diabetics. The PROC SURVEY procedure was utilized to account for multistage sampling and weights. All analysis was performed in SAS 9.3 [15].

## Results

A total of 36, 489 adults with diabetes aged 19–60 years were surveyed and provided valid data for the analysis. 50% of participants were female, 79% were of white race, 16.4% were of black race, and 3.9% were of Asians race (p<0.05); mean age (±SEM) was 45.7 years (±0.02). The overall rate of hepatitis B vaccination among adults 19–60 years old (population in whom Hepatitis B vaccination is recommended) was 20.2%. The rate of vaccination differed across racial groups (Asians 26.0% vs. blacks 23.9%; and whites 19.4%; p<0.01). Diabetics at least 40 years and older (25.3% vs. 40.4%), who did not graduate college (19.0% vs. 27.8%), or were foreign born (19.3% vs. 20.6%) also had lower vaccination rates than their corresponding groups (Table 1). In addition, individuals without health insurance coverage had lower vaccination rates (20.1% vs. 24.0%); individuals with stroke had very low vaccination rates (15.1% vs. 21.0%, p<0.01), while individuals with failing kidneys had high vaccination rates (25.9% vs. 20.1%, p<0.01). The unadjusted odds ratio (OR) for hepatitis B vaccination was 1.46 (95% CI=1.36–1.57, p<0.01) for diabetic Asians, and 1.31 (95% CI=1.19–1.44, p<0.01) for diabetic blacks; (Table 2); both when compared to diabetic Whites. Multivariate logistic regression showed that the adjusted (for age, BMI and college education) OR for hepatitis B vaccination was 1.36 (95% CI=1.29–1.43, p<0.01) for diabetic Asians, and 1.36 (95% CI=1.19–1.44, p<0.10) for diabetic blacks; both when compared to diabetic Whites.

## Conclusion

The higher hepatitis B vaccine utilization was more marked among Asians, and Blacks, when compared to Whites. The disparity in coverage was more substantial for individuals who were non-citizens, recent immigrants, or non-English speakers. Our data showed that Hispanics, and foreign-born individuals also had lower vaccination rates. Additionally, individuals without a college level degree or health insurance coverage also had lower rates of utilization.

## Discussion

Our results show low hepatitis B vaccination rates among patients enrolled in the study, despite CDC recommendation for vaccination for all adult patients under age 60 [16]. Furthermore, vaccination rates for individuals with diabetes differ among racial, demographic, medically co-morbid categories. The racial disparity in hepatitis B vaccination rate seen in our study may be consistent with the historically high hepatitis B prevalence recorded in the Asian and African American populations, predisposing to relative cultural awareness of their risk profile. Additionally, physicians may be more vigilant in monitoring the aforementioned populations due their increased risk of hepatitis B, thereby increasing the vaccination rates compared to other populations. However, further vigilance is necessary on the part of health-care workers for promoting Hepatitis B vaccine coverage, as coverage rates fall far short of the blanket recommendation for vaccination. Culturally sensitive vaccination programs have been previously effective in increasing vaccination rates amongst high risk patient populations, and may need to be implemented to improve adherence to vaccination recommendations amongst patients with diabetes. For example, a past CDC survey showed hepatitis B vaccine coverage to be 41%–61% and 2%–11% for cities with and without vaccination programs for Asian Pacific Islander children, respectively [16].

Lu et al., showed lower hepatitis B vaccination coverage rates among foreign born individuals compared to those who were US born [17]. This is consistent with the findings of our study. This may be due in part to economic emigrational forces and globalization affecting highly endemic regions of Southeast and Far East Asia [18]. PJ and colleagues also recognized the need for more culturally sensitive educational outreach efforts which promote awareness for HBV screening, prevention, and treatment within a community with high foreign born non English speakers [17]. PJ suggests the delivery of health care in native languages and increased awareness in the primary care community-based clinics as strategies which can be implemented to improve vaccination rates.

Our data shows college education and health insurance status, commonly used surrogates for socioeconomic status, were correlated with vaccination utilization. This is consistent with other studies that have shown differences in hepatitis B vaccination status related to socioeconomic status. Namely, important factors affecting hepatitis B coverage include household income level, education, older age and access to care [19,20].

Finally, it is important to note that the CDC recommendations [21], for vaccinations of adults with diabetes had been reaffirmed by the most recently published 2017 Standards of Medical Care in Adults with Diabetes by the American Diabetes Association [22].

We also like to mention that the lower rate of vaccination among whites, and the higher rates of vaccination among obese could be explained by the higher rate of co-morbidity and disease severity index among minorities and obese patients bringing these individuals more frequently to medical attention and leading to more opportunities for vaccinations.

## Figures and Tables

**Table 1:** Hepatitis B vaccine rates among ethnic and demographic groups of Adult Diabetic individuals.

	Vaccination (%)	No Vaccination (%)	p-value
All survey participants	28.6	71.4	0.01
Asians	26.0	74.0	0.01
Blacks	23.9	76.1	
Whites	19.4	81.6	
Hispanic	19.0	81.0	0.01
Non-Hispanic	20.6	79.4	
Female	22.1	77.9	0.01
Male	18.8	81.2	
Age 19–39 years	40.4	59.6	0.01
Age 40–60 years	25.3	74.7	
Foreign Born	20.6	79.4	0.01
US Born	80.7	19.3	
No College Degree	19.0	81.0	
College Degree	27.8	72.2	0.01
Current/Former Alcohol use	29.2	70.8	0.01
Lifetime abstainers	23.4	76.6	
No Insurance	24.0	76.0	0.01
Insurance	20.1	79.9	
Normal weight (BMI 18.5–24.99)	14.5	17.5	0.01
Overweight (BMI 25–29.99)	29.6	32.9	
Obese (BMI ≥ 30)	55.9	49.6	
Cancer	17.0	83.0	0.01
No Cancer	21.0	79.0	
Weak/Failing Kidneys	25.9	74.1	0.01
No Weak/Failing Kidneys	20.1	79.9	
Stroke	15.1	84.9	0.01
No Stroke	21.0	79.0	
Heart Failure	13.3	86.7	0.05
No Heart Failure	17.5	82.5	

**Table 2:** Ratio of hepatitis B vaccination rates among adult individuals with diabetes by demographics.

	Odds Ratio	95% Confidence Interval	p-value
Asians Unadjusted	1.46	1.36–1.57	0.01
Blacks Unadjusted	1.31	1.19–1.44	0.05
Whites (ref)	1.00	1.00	n/a
Asians Adjusted	1.36	1.29–1.43	0.01
Blacks Adjusted	1.36	1.10–1.68	0.01
Whites (ref)	1.00	1.00	n/a
Hispanic	0.80	0.26–2.42	0.69
Female	1.69	1.33–2.15	0.01
Age 41–60 years (ref 19–40 years)	0.46	0.41–0.51	0.01
Foreign Born	1.33	0.54–3.44	0.56
Married/living partner	1.21	0.93–1.56	0.15
Lack of College Degree	0.63	0.53–0.74	0.01
Alcohol	1.34	1.16–1.55	0.01
Overweight	0.81	0.31–2.11	0.44
Obese	0.94	0.35–2.49	0.86
Normal weight (reference)	1.0	1.0	n/a
Have Insurance	1.19	0.68–2.09	0.53
Cancer	0.92	0.91–0.94	0.01
Weak/Failing Kidneys	1.562	1.43–1.70	0.01
Stroke	0.18	0.15–0.21	0.01
Heart Failure	1.28	0.95–1.70	0.10
